# Flexible modeling improves assessment of prognostic value of C-reactive protein in advanced non-small cell lung cancer

**DOI:** 10.1038/sj.bjc.6605603

**Published:** 2010-03-16

**Authors:** B Gagnon, M Abrahamowicz, Y Xiao, M-E Beauchamp, N MacDonald, G Kasymjanova, H Kreisman, D Small

**Affiliations:** 1 Department of Medicine and Oncology, McGill University, 687 Pine Avenue West, R4.29, Montreal, Quebec, H3A 1A1, Canada; 2 Department of Epidemiology and Biostatistics, McGill University, 687 Pine Avenue West, V2, Montreal, Quebec, H3A 1A1, Canada; 3 Department of Oncology, Cancer Nutrition and Rehabilitation Program, McGill University, 687 Pine Avenue West, Montreal, Quebec, H2A 1A1, Canada; 4 Department of Internal Medicine, Division of Pulmonary Diseases, Sir Mortimer B Davis, Jewish General Hospital, 3755 Cote-Ste-Catherine, Montreal, Quebec, H3T 1E2, Canada; 5 Departments of Oncology and Internal Medicine, Division of Pulmonary Diseases, Sir Mortimer B Davis, Jewish General Hospital, 3755 Cote-Ste-Catherine, Montreal, Quebec, H3T 1E2, Canada

**Keywords:** non-small cell lung cancer, inflammation, prognosis, C-reactive protein, albumin, survival analysis

## Abstract

**Background:**

C-reactive protein (CRP) is gaining credibility as a prognostic factor in different cancers. Cox's proportional hazard (PH) model is usually used to assess prognostic factors. However, this model imposes *a priori* assumptions, which are rarely tested, that (1) the hazard ratio associated with each prognostic factor remains constant across the follow-up (PH assumption) and (2) the relationship between a continuous predictor and the logarithm of the mortality hazard is linear (linearity assumption).

**Methods:**

We tested these two assumptions of the Cox's PH model for CRP, using a flexible statistical model, while adjusting for other known prognostic factors, in a cohort of 269 patients newly diagnosed with non-small cell lung cancer (NSCLC).

**Results:**

In the Cox's PH model, high CRP increased the risk of death (HR=1.11 per each doubling of CRP value, 95% CI: 1.03–1.20, *P*=0.008). However, both the PH assumption (*P*=0.033) and the linearity assumption (*P*=0.015) were rejected for CRP, measured at the initiation of chemotherapy, which kept its prognostic value for approximately 18 months.

**Conclusion:**

Our analysis shows that flexible modeling provides new insights regarding the value of CRP as a prognostic factor in NSCLC and that Cox's PH model underestimates early risks associated with high CRP.

Patients with advanced non-small cell lung cancer (NSCLC) have a grim prognosis; most will die in less than a year (Schrump *et al*, 2008). Identifying patients at higher risk of very short survival times is important for optimal clinical management. Furthermore, the patients’ understanding of their prognosis influences their willingness to receive life-extending therapy (Weeks *et al*, 1998a). Yet, estimation of expected survival times remains a clinically difficult (Glare, 2005; Watine *et al*, 2006; Kellett, 2008) and an emotionally challenging task (Berry, 2008).

In recent years, different biological markers have been suggested to improve prognostication of cancer (Mahmoud and Rivera, 2002; Maltoni *et al*, 2005). Among them, inflammatory markers, especially C-reactive protein (CRP), have been suggested to predict survival in different cancers (McMillan, 2008; Koch *et al*, 2009), including NSCLC (Kato *et al*, 2000; Forrest *et al*, 2003, 2004). In these published studies of NSCLC mortality (Kato *et al*, 2000; Forrest *et al*, 2003, 2004), the prognostic value of CRP was assessed using the Cox's proportional hazard (PH) model (Cox, 1972).

To improve both prognosis and clinical management, an accurate assessment of the independent relationships between putative prognostic factors, such as CRP, and mortality is paramount. To ensure valid conclusions and accurate risk prediction, prognostic studies should rely on statistical methods that correctly represent the actual structure of empirical data and the true complexity of the biological processes under study. From this perspective, it is imperative to verify the assumptions underlying the statistical models to be used in the analysis (Altman *et al*, 1995). The Cox's PH model imposes the assumption that the mortality hazards associated with different patterns of covariates (‘risk profiles’) are proportional, which implies that the estimated effects of prognostic factors on the hazard are *a priori* constrained to remain constant over the entire follow-up time (Cox, 1972). This crucial assumption is rarely tested in prognostic studies of cancer mortality (Altman *et al*, 1995). Yet, in different cancers, it has been shown to be inconsistent with the actual effect of various prognostic factors, whose effect on mortality did change over time (Gray, 1992; Hess, 1994; Kooperberg *et al*, 1995; Rachet *et al*, 1998; Quantin *et al*, 1999; Lambert *et al*, 2005; Remontet *et al*, 2007; Binquet *et al*, 2009). In such situations, an inappropriate use of the Cox's PH model may lead to biased results, inaccurate prediction, loss of statistical power, and incorrect conclusions (Altman *et al*, 1995; Abrahamowicz *et al*, 1996).

Furthermore, conventional statistical models, such as the Cox's PH model, rely on the linearity assumption, according to which the relationships between continuous prognostic factors and the respective outcome measure (logarithm of the hazard in the case of Cox's PH model) are linear. This would imply that, for example, the relative risk of mortality is the same when comparing (a) an 80-year old *vs* a 60-year-old subject, and (b) a 40-year old *vs* a 20-year old, because in both cases there is a 20-year age difference. Again, in the last two decades, several epidemiological and clinical studies have shown that the linearity assumption is seriously violated for many prognostic and risk factors, and its *a priori* acceptance may lead to important biases and misleading conclusions (Hastie and Tibshirani, 1990; Sleeper and Harrington, 1990; Gray, 1992; Royston and Altman, 1994; Abrahamowicz *et al*, 1997; Remontet *et al*, 2007; Royston and Sauerbrei, 2008). Thus, the methodological arguments and the empirical evidence indicate that both the PH and the linearity assumptions should be carefully verified in prognostic studies.

Our aim is to re-assess the ability of CRP to predict survival in a cohort of patients recently diagnosed with non-resectable NSCLC before receiving chemotherapy. To this end, we used the flexible generalisation of the Cox's model, which permits testing the conventional PH and linearity hypotheses, and avoids imposing the *a priori* assumptions underlying the Cox's PH model. If these hypotheses were rejected, non-proportional and/or non-linear effects of particular prognostic factors were estimated (Abrahamowicz and MacKenzie, 2007).

## Patients and methods

### Patients

Since May 2001, clinical data of patients with lung cancer, observed at the Jewish General Hospital Pulmonary Oncology Clinic (POC) in Montreal, Quebec, Canada, were prospectively recorded in a computerised database. These data included, among other characteristics, age, sex, stage, ECOG performance status (Oken *et al*, 1982), smoking status, type of first-line chemotherapy regimen, and the blood parameters listed in Table 1. The level of CRP started to be measured in January 2002 by one POC physician and, after January 2005, by all POC physicians. A majority of the blood tests were taken within 3 days before the first chemotherapy dose.

For the purpose of this study, we extracted the data for patients treated between 9 April 2002 and 18 September 2008, and terminated their follow-up on 15 March 2009. Dates of death were obtained from clinical charts. The study was approved by the Institutional Review Board of the Jewish General Hospital.

### Statistical analyses

#### Data analyses

Descriptive statistics were used to compare the baseline characteristics of subjects for whom CRP was available and who were, thereby, included in the analyses, *vs* those excluded.

In the main analyses, we used multivariable survival analytical methods for right-censored time-to-event data (Cox, 1972). Time 0 was defined as the date of the first chemotherapy treatment and the event of interest was death because of any cause. Patients who were alive at the end of the study, on 15 March 2009, were censored. In preliminary analyses, we assessed the distributions of continuous variables and used a logarithmic transformation with base 2 for the only two variables with considerable positive skewness: CRP and lactate dehydrogenase (LDH).

#### Statistical models

Two different types of survival analytical models were used. First, we used both the univariate and the multivariable Cox's PH models (Cox, 1972), which *a priori* imposed the PH assumption and, for continuous covariates, the linearity assumption.

The second model represented a flexible generalisation of the Cox's model, recently developed and validated by Abrahamowicz and MacKenzie (Abrahamowicz and MacKenzie, 2007). The general idea of flexible modeling is to avoid restrictive *a priori* assumptions underlying standard statistical models and model the effects of prognostic factors through flexible functions, the shapes of which are estimated directly from the data, rather than being imposed by the model. Such flexible models use various, typically computationally intensive, statistical techniques, such as regression splines (Ramsay, 1988), generalised additive models (Hastie and Tibshirani, 1990), or fractional polynomials (Royston and Sauerbrei, 2008). The flexible model used in our analyses uses quadratic regression splines, that is, piecewise quadratic polynomials that can recover a large variety of clinically plausible functions and, thus, accurately estimate both non-PH and non-linear effects of continuous predictors (Abrahamowicz and MacKenzie, 2007). Throughout the paper, we use the term ‘flexible spline-based model’ to refer to the flexible generalisation of the Cox's PH model that uses quadratic regression splines to model time-dependent and/or non-linear covariate effects with quadratic regression splines, as in (Abrahamowicz and MacKenzie, 2007).

The flexible spline-based model allowed us to test, for each continuous prognostic factor, the two assumptions underlying the Cox's PH model: (a) the PH hypothesis that the effect of the predictor remained constant over time and (b) the linearity of the effect on the log hazard (linearity hypothesis). If one or both of these assumptions were rejected for a given prognostic factor, then the flexible spline-based model permitted an accurate re-modeling of its effect on the hazard. Specifically, if the PH hypothesis was rejected, with *P*<0.05 for a non-parametric model-based likelihood ratio test (LRT), then splines were used to model the time-dependent hazard ratio (HR), that is, to estimate how the strength of the predictor's effect on the hazard changes with increasing follow-up time (Abrahamowicz and MacKenzie, 2007). Conversely, if the linearity hypothesis was rejected, then splines were used to assess how the risk (log hazard) changed with increasing predictor value. If these hypotheses were not rejected (*P*>0.05), then, to avoid over-fit bias (Abrahamowicz and Ciampi, 1991) and adhere to the model parsimony principle, the predictor effect was estimated as, respectively, constant-over-time HR and/or linear function. For binary predictors, only the PH hypothesis was tested and, if rejected, the time-dependent effect was estimated (Abrahamowicz *et al*, 1996).

#### Univariate analyses

The analyses started with a series of separate, univariate Cox's PH models, each evaluating one of the following baseline characteristics, initially considered as potential prognostic factors for NSCLC mortality (Table 1): (a) categorical variables: sex, stage (IIIA and IIIB without pleural effusion *vs* IIIB with pleural effusion and IV), performance status (ECOG 0–1 *vs* 2), smoking history (ever *vs* never), chemotherapy regimen (double *vs* single agents) and tumour pathology, and (b) continuous variables: age, log-transformed CRP (log_2_ CRP), albumin, log-transformed LDH (log_2_ LDH), calcium, alkaline phosphatise (ALP), haemoglobin, platelet, absolute neutrophil count (ANC), lymphocyte count, and percentage of weight lost.

Next, we estimated a series of univariate flexible spline-based models, each for a single prognostic factor, which tested the PH and, for continuous variables, the linearity assumptions, without adjustment for the other variables.

#### Strategies for building multivariable models

**Figure 1:**
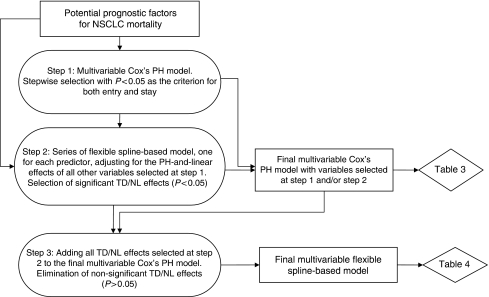
Flowchart of multivariable models building.

Building a flexible multivariable model is challenging, especially if potential predictors are correlated (Sauerbrei *et al*, 2007). To build parsimonious final multivariable models, we used a multi-step procedure (Binquet *et al*, 2008). The flow chart in Figure 1 illustrates the consecutive steps of the analyses, explains how the results of the earlier steps affected the later steps, and identifies which results are presented in which table.

(1) At the first step, we first included all the aforementioned variables, listed in Table 1, in the multivariable Cox's PH model and then used the stepwise selection procedure, with *P*<0.05 and *P*>0.05 for the two-tailed Wald test as the criteria for, respectively, variables inclusion and elimination from the model. The selected variables were included in the final multivariable Cox's PH model. In addition, we have forced into the final Cox's PH model the performance status (ECOG 0–1 *vs* 2), even if it has a marginally nonsignificant effect in our analyses (*P*=0.086), because it is an essential criterion for the treatment of unresectable NSCLC (Pfister *et al*, 2004).

(2) The next step of the multivariable model building process accounted for the fact that a variable may not be a significant predictor of survival in the Cox's PH model, which imposes *a priori* the PH and linearity assumptions, but may still have a significant time-dependent or non-linear effect (Abrahamowicz and MacKenzie, 2007). Therefore, the second step of the model building process involved fitting a series of separate flexible spline-based models, each of which tested the time-dependent and, for continuous variables, non-linear effects of a single variable, while adjusting for the PH-and-linear effects of all other variables selected at step 1 (Binquet *et al*, 2008). In other words, each model fitted at step 2 expanded the multivariable Cox's PH model built at step 1 by estimating and testing time-dependent and non-linear effects of a single predictor. On the basis of the results of the tests carried at the first and the second steps, we identified those variables that had statistically significant effects (*P*<0.05) in at least one of the following: (a) Wald test of the association in the multivariable Cox's PH model selected by the stepwise selection in step 1, and/or (b) non-parametric LRT's of time-dependent and/or non-linear effect(s) in the respective flexible spline-based model, estimated at step 2. Only those variables that met criteria (a) and/or (b) were included in the final multivariable versions of both Cox's PH model and the flexible spline-based model.

(3) To build the final multivariable flexible spline-based model, we first expanded the final Cox's PH model by including all ‘non-parametric’, that is, time-dependent and/or non-linear, effects that were statistically significant (*P*<0.05) at step 2. We then eliminated all those ‘non-parametric’ effects that became nonsignificant (*P*>0.05) when adjusted for other ‘non-parametric’ effects as well as for all variables selected, and forced into the multivariable model, at steps 1 and 2. As a result, the final multivariable flexible spline-based model included only those time-dependent and non-linear effects that remained statistically significant even when adjusted for each other, while all other variables selected for both final multivariable models were represented by parametric constant-over-time (PH) linear effects (Binquet *et al*, 2008).

In the final flexible spline-based model, we tested the overall statistical significance of the effects of those continuous variables, which were represented by both non-linear and time-dependent functions. To this end, we used the 5-degree-of-freedom (df) non-parametric LRT that compared the deviance of the final flexible model with that of the corresponding reduced flexible model, from which a given variable was completely eliminated (Abrahamowicz and MacKenzie, 2007). The resulting *P*-value indicated the overall statistical significance of the independent association between the hazard and the variable, after having accounted for its non-linear and time-dependent effects.

The goodness-of-fit of the Cox's PH model *vs* its flexible spline-based extension was compared with the Akaike's information criterion (AIC) (Akaike, 1974), which accounted for the increased complexity of the flexible spline-based model. A decrease of 10 or more AIC points indicates an important improvement in the model's predictive ability (Quantin *et al*, 1999).

#### Model validation

To further compare the predictive ability of our final flexible spline-based model with that of the Cox's PH model, with the same predictors, we relied on the ‘internal’ cross-validation procedure (Harrell, 2001). Specifically, we used the five-fold cross-validation algorithm (Rachet *et al*, 2003; Binquet *et al*, 2008), which involved splitting our sample of 269 patients into five randomly selected, mutually exclusive subsets of equal size. Then, the following two steps were repeated five times, separately for the Cox's PH and the flexible spline-based model. (1) One of the five subsets (validation subsample) was left out and the model was estimated using data from only the four other subsets. (2) The regression coefficients from step 1 were used to calculate the partial deviance for the respective validation subsample, that is, to assess how well the outcomes in that subsample were predicted by a given model (Binquet *et al*, 2008). Finally, the five deviance values, each from a different subset, were summed up to obtain the cross-validated partial deviance of the entire data set. As each subset-specific deviance was calculated based on the model that did *not* depend on the data in the corresponding validation subset, the lower cross-validated deviance indicated the model expected to better predict the outcomes in an independent data set from a similar population (Rachet *et al*, 2003; Binquet *et al*, 2008).

Descriptive analyses and conventional Cox's PH regression were performed using the SAS statistical package (SAS Institute Inc., Cary, NC, USA), while the flexible spline-based model was implemented with a customised programme (Abrahamowicz and MacKenzie, 2007) written in the C programming language (Abrahamowicz *et al*, 1996).

## Results

### Patients’ characteristics

The CRP level was determined in 64 (39%) among 163 patients diagnosed with NSCLC who received chemotherapy between April 2002 and January 2005, and in all 220 consecutive patients between January 2005 and 15 September 2008. In all, 13 patients were excluded because of missing covariate values and 2 because of outlier values for platelet (<40 000 × 10^−6^ l^–1^). Therefore, 269 (70%) out of 383 patients were available for the analyses. Table 1 presents baseline characteristics of the patients included in the study. For all 269 study subjects, we had complete data on all covariates shown in Table 1, with the exception of five (2%) patients, who had missing data on calcium. Excluded patients had similar characteristics, except that they received single-agent regimen of chemotherapy more frequently than the patients included in the analyses (results not shown).

The minimum follow-up was 3 days (acute complications after initiation of chemotherapy), with a median follow-up of 8.6 months and median survival of 9.2 months. During the follow-up, 211 (78.4%) patients died. No patients were lost to follow-up.

### Univariate survival analyses and multivariable model building

Left part of Table 2 summarises the results of separate, univariate Cox's PH models, each evaluating one of the potential predictors. The third column of Table 2 shows that in the univariate Cox's PH models, all variables, with the exception of age and tumour pathology, had statistically significant (*P*<0.05) or marginally significant (0.05<*P*<0.10) crude unadjusted associations with the hazard. However, the two last columns show that, in univariate flexible spline-based models, either the PH or the linearity assumptions were violated (*P*<0.05 for the respective test) for several variables.

In the multivariable Cox's PH regression analyses, the stepwise selection procedure eliminated age, sex, tumour pathology, calcium, haemoglobin, platelet, and percentage of weight lost, as their adjusted PH-linear effects were all statistically nonsignificant (*P*>0.05). In the multivariable Cox's PH model, the constant-over-time, linear effects of albumin and ALP were also nonsignificant (third column of Table 3). However, both variables were kept in the final multivariable models because of their significant effects in respective flexible spline-based models, estimated at the step 2 of the model building process (see ‘Statistical analyses’). As shown in the two last columns of Table 3, while adjusted for other predictors selected into the final multivariable models, both time-dependent (*P*<0.001) and non-linear (*P*=0.024) effects of albumin, as well as the non-linear effect of ALP (*P*=0.034), were all significant.

### Final multivariable Cox's PH model

The left part of Table 3 summarises the results of the final multivariable Cox's PH model. C-reactive protein was found to be a very significant independent predictor of survival, with a 11% increase in the risk of death for each doubling of its value (adjusted HR=1.11, 95% CI: 1.03–1.20, *P*=0.008). Among binary prognostic factors, smoking (ever *vs* never), higher baseline cancer stage (IIIB+pleural effusion *vs* IIIA/IIIB), and type of chemotherapy regimen (single- *vs* double-agent regimen) were all independently associated with significantly higher mortality, while higher performance status (ECOG 2 *vs* 0–1) showed a trend toward worse prognosis (*P*=0.086). In addition to CRP, other significant continuous predictors were LDH, with more than a two-fold, 116% increase in the risk of death for each doubling of its value, lower lymphocyte count, and higher ANC (third column of Table 3). In contrast to the univariate Cox's PH model (third column of Table 2), in the final multivariable Cox's PH model, albumin was not a significant predictor of mortality (HR=1.02, 95% CI: 0.97–1.06, *P*=0.485).

### Final flexible spline-based multivariable model

At the step 3 of the model building process, we first built a large flexible spline-based model that included all ‘non-parametric’ (time-dependent and/or non-linear) effects that were identified as significant (*P*<0.05) in either of the two rightmost columns of Table 3. After having adjusted for all other non-parametric effects included in this large model, both the time-dependent and the non-linear effects of ANC, as well as the non-linear effect of ALP, became nonsignificant (data not shown). Accordingly, all three effects were eliminated from the final flexible spline-based model. In contrast, the time-dependent and non-linear effects of both albumin and log_2_ CRP retained their statistical significance and, thus, were kept in the final flexible model.

Table 4 summarises the results of the final flexible spline-based multivariable model, with the same predictors as the multivariable Cox's PH model shown in Table 3. In the final flexible spline-based model, only log_2_ CRP and albumin had significant non-parametric effects. For the eight other covariates, the adjustment for non-linear and time-dependent effects of log_2_ CRP and albumin do not materially alter the HRs, relative to the Cox's PH model, and all conclusions regarding their statistical significance are the same in both final multivariable models (Table 3 *vs* Table 4).

For log_2_ CRP and albumin, the last column of Table 4 shows *P*-values for the 5-df tests of the overall statistical significance of their adjusted effects on the hazard. Both *P*-values are below 0.01, indicating that, after having accounted for their non-linear and time-dependent effects, both variables have highly significant associations with mortality. For albumin, this finding is striking in contrast to its completely nonsignificant effect in the multivariable Cox's PH model (*P*=0.485 in Table 3).

**Figure 2:**
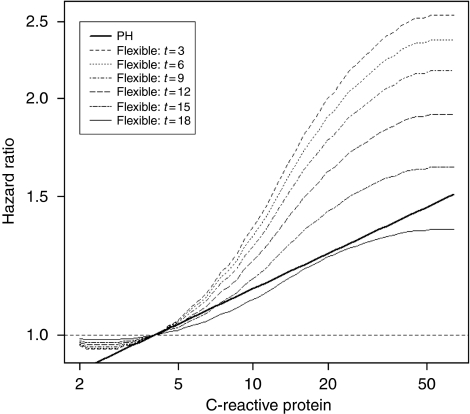
Results of the Cox's PH and flexible spline-based multivariable modeling of the effect of CRP on survival. The bold line represents the linear estimate from the Cox’s PH model. The curves correspond to the flexible spline estimates at different times from 3 months (*t*=3) to 18 months (*t*=18) after the initiation of chemotherapy. Each curve shows how the adjusted hazard ratio at the corresponding time, relative to the value of 4 mg l^−1^, changes with increasing value of C-reactive protein.

In the final flexible spline-based multivariable model, log_2_ CRP had a statistically significant time-dependent effect, as the PH assumption was rejected (*P*=0.033), and a significant non-linear relationship with the logarithm of the mortality hazard (linearity rejected at *P*=0.015). Figure 2 shows the implications of the joint violation of the PH and linearity assumptions for the predictive ability of baseline CRP. The thick black line in Figure 2 shows the linear, constant-over-time effect of increasing the baseline CRP, as estimated in the multivariable Cox's PH model. As the Cox's model imposes the PH assumption, this linear effect is assumed to apply to all times during the follow-up. In contrast, the dashed curves in Figure 2 represents the effect of baseline CRP estimated in the flexible spline-based model, for different times elapsed because the CRP was measured. Each curve shows how the current risk of all-cause mortality, at that specific follow-up time, changes with the increasing value of the baseline CRP. The fact that the curves rise steeply confirms that higher CRP is associated with an important risk increase. However, the slope of the curves differs across different intervals of CRP values: the risk increases are much steeper between 5 and 30 mg l^–1^ than outside this interval (Figure 2), which reflects the important non-linear effect of CRP. The steep increases in mortality in the middle range of CRP values indicate also that any dichotomisation of these values, regardless of the potential cut-off, will entail an important loss of information, as patients with substantially different actual risks will be predicted to have the *same* risk. For example, at 3 months of follow-up, CRP=50 mg l^–1^ is associated with an almost two-fold risk increase relative to CRP=11 mg l^–1^ (the top curve in Figure 2), even if both values are above the conventional CRP cut-off of 10 mg l^–1^.

The curves in Figure 2 become gradually less steep as the follow-up time increases. This reflects the significant time-dependent effect of CRP and indicates that the effect of high baseline CRP on mortality gradually decreases with increasing time since its initial measurement. Indeed, Figure 2 suggests that the baseline CRP retains some predictive value only for the initial 12–18 months. In sensitivity analyses, we investigated the potential time-dependent effect of the dichotomised CRP, with the conventional >10 mg l^–1^ cut-off (Mahmoud and Rivera, 2002; Maltoni *et al*, 2005; McMillan, 2008). As in the primary analyses, with the non-linear effect of continuous CRP, the PH hypothesis was rejected for binary CRP (*P*=0.035). Furthermore, the time-dependent estimate for the dichotomised CRP was very similar to that for continuous CRP, with a gradual decrease of its effect over time (data not shown). In the first 8 months after the initiation of chemotherapy, patients with baseline CRP>10 mg l^–1^ had a two-fold higher mortality than patients below the cut-off, with the same values of all other prognostic factors, but 2 years after diagnosis their relative risk increase was as small as 20% (data not shown).

**Figure 3:**
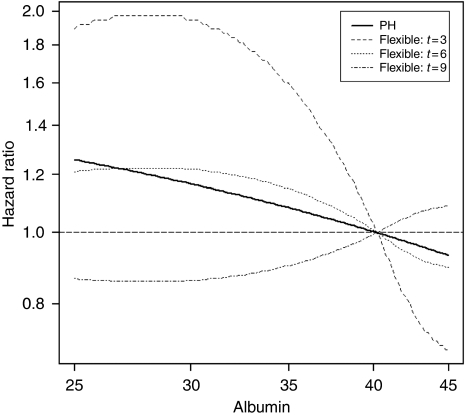
Results of the Cox's PH and flexible spline-based multivariable modeling of the effect of albumin on survival. The bold line represents the linear estimate from the Cox’s PH model. The curves correspond to the flexible spline estimates at different times from 3 months (*t*=3) to 9 months (*t*=9) after the initiation of chemotherapy. Each curve shows how the adjusted hazard ratio at the corresponding time, relative to the value of 40 mg l^−1^, changes with decreasing value of albumin.

In the final flexible spline-based model, albumin also had significant non-linear (*P*=0.038) and, especially, time-dependent effects (*P*<0.001). The three dashed curves in Figure 3, constructed similarly to Figure 2, show how the hazard of mortality changes with increasing value of baseline albumin, respectively, at 3, 6, and 9 months of follow-up. At 3 months, patients with low initial albumin, between 25 and 30 mg l^–1^, have approximately a two-fold higher risk of death than those with the sample mean value of approximately 40 mg l^–1^ (the steepest curve, at the top of Figure 3). In contrast, by 9 months of follow-up the curve becomes very flat, indicating that initial albumin value has no predictive value at or beyond 9 months after it was measured (the curve at the bottom of Figure 3). This sharp decrease in the prognostic value of baseline albumin during the follow-up reflects its very significant time-dependent effect (*P*<0.001). This also explains why albumin was completely nonsignificant in the multivariable Cox's PH model (*P*=0.485), in which its estimated effect was *a priori* constrained to be constant over time. By imposing this constraint, totally inconsistent with the actual, very significant time-dependent effect of albumin, the Cox's PH estimate, represented by a thick black line in Figure 3, suggested decreasing albumin had only a very weak effect on increased mortality hazard. This masked the important short-term increase in risk for patients with low baseline albumin, below 35 mg l^–1^, shown by the flexible spline estimate, for *t*=3 months, in Figure 3.

### Goodness-of-fit and model validation

The final flexible spline-based multivariable model, which accounted for the significant time-dependent and non-linear effects of both log_2_ CRP and albumin, yielded an important improvement in the fit to our data, as reflected by the much lower value of AIC than for the multivariable Cox's PH model, with the same variables (AIC=1909.3 in Table 4 *vs* 1922.2 in Table 3).

The five-fold cross-validation confirmed that the flexible spline-based multivariable model could be expected to better predict the relative risks in an independent sample from a similar population, as its cross-validated deviance was substantially lower than for the Cox's PH model with the same predictor variables (1230.5 *vs* 1263.6).

## Discussion

We have re-assessed the role of the CRP and other biomarkers in the prognosis of NSCLC. We considered a larger number of potential prognostic factors (Table 1) than most previous studies of NSCLC mortality (Brundage *et al*, 2002; Watine *et al*, 2006) and tested the important assumptions underlying the Cox's PH model, on which those studies relied (Kato *et al*, 2000; Forrest *et al*, 2003, 2004). To this end, we have used a new, flexible spline-based model that permitted testing of the conventional PH and linearity assumptions, and accounting for their violations (Abrahamowicz and MacKenzie, 2007). The results confirmed the advantages of such flexible modeling by revealing statistically and clinically significant violations of both assumptions for CRP and albumin. Below, we explain in detail the important clinical implications of accounting for these violations for the prognosis of individual patients survival in NSCLC.

The multivariable Cox's PH model in Table 3 suggested that the risk of mortality increased by approximately 11% for each doubling of CRP, and the underlying linearity assumption would imply that the same increase applied to the comparisons of CRP of (a) 4 *vs* 8 mg l^–1^, as (b) 8 *vs* 16 mg l^–1^. Yet, our flexible spline-based model indicated that the linearity assumption was violated (*P*=0.015), and that the actual risk increase was much steeper between CRP values of 8 and 16 mg l^–1^ (Figure 2). A majority of the published cancer prognostic studies dichotomise CRP at 10 mg l^–1^ (Mahmoud and Rivera, 2002; Maltoni *et al*, 2005; McMillan, 2008), and the Glasgow Prognostic Score uses the same cut-off (Forrest *et al*, 2003). Yet, Figure 2 shows that while patients with CRP>10 mg l^–1^ are, on average, at much higher risk than those below this cut-off, the risk of death increases continuously between 4 and 50 mg l^–1^, with steepest increases between 8 and 20 mg l^–1^. Consequently, at 3 months of follow-up, CRP=50 mg l^–1^ is associated with almost two-fold higher risk than CRP=11 mg l^–1^, even if both values fall above the conventional 10 mg l^–1^ cut-off. Thus, our flexible, non-linear estimate of the CRP effect helps avoiding inaccurate risk assessment and loss of prognostic information, which would be induced by conventional dichotomised or linear estimates (Ramsay, 1988; Hastie and Tibshirani, 1990; Royston and Altman, 1994; Greenland, 1995; Abrahamowicz *et al*, 1997; Benedetti and Abrahamowicz, 2004).

Second, the PH assumption imposes that in the Cox's PH model the relative risk associated with each prognostic factor remains constant during the entire follow-up period. Thus, the HR between any two baseline CRP values is *a priori* constrained to be the same at the time of its measurement as, for example, 12 months later. However, our flexible spline-based analyses rejected the PH hypothesis for CRP, and indicated that it retains the prognostic value for approximately a year (Figure 2). The gradual loss of prognostic ability of the baseline CRP may occur because (a) some patients, with high initial CRP value, for unknown reasons, responded to chemotherapy better than others, (b) in patients with oesophageal squamous cell cancer, CRP polymorphism may gradually modify tumour progression (Motoyama *et al*, 2009), and/or (c) correlation between the baseline CRP and its current values decreases over time. Future studies should examine the latter conjecture, by using time-dependent covariates to model the effect of updated CRP values.

For albumin, our flexible spline-based model revealed a dramatic decrease in its prognostic value over time (*P*<0.001). Figure 3 shows that low baseline albumin, below 30 mg l^–1^, is associated with a statistically significant and clinically important mortality risk increase in the first 3 months of follow-up. However, the effect of baseline albumin on mortality rapidly declines thereafter, and becomes practically null after approximately 9 months. This rapid loss of prognostic value explains why albumin was completely nonsignificant in the multivariable Cox's PH model (*P*=0.485), which estimates the average relative risks across the follow-up (Abrahamowicz *et al*, 1996).

In conclusion, the Cox's model, by imposing the incorrect PH assumption, failed to identify albumin as an important early prognostic factor for NSCLC mortality. Clinical observations suggested that low albumin might indicate an unfavourable prognosis, especially in the near future (Nixon *et al*, 1980; Hill, 1987; Heys *et al*, 1992). Still, our flexible spline-based model showed the statistical significance of time-dependent changes in the effect of albumin, and detected the dramatic effect of low baseline albumin on NSCL mortality in the next 3–4 months. Thus, flexible analyses enhanced both the validity and the accuracy of conclusions regarding prognostic value of albumin.

The implications of the violation of the conventional PH and linearity assumptions are relevant for clinicians. By accounting for the time-dependent changes in the effects of both markers, the flexible model helps them to realise that the risk of death associated with high CRP and low albumin is very high for the first 6–12 months after diagnosis. Furthermore, by accounting for important non-linearities of the relationships between the marker values and the hazard, the flexible model improves substantially clinicians’ ability to identify high-risk subgroups. For example, the Cox's PH model incorrectly suggests that only a small increase (23%) of risk of death occurs with an increase of baseline CRP from 4 to 16 mg l^–1^, while in the more accurate, flexible model it is associated with a much higher (80%) risk increase. Such objective prognostic information, when provided in the timely manner, may influence patients’ crucial decisions, possibly making them more likely to decline life-prolonging therapy and to opt for comfort care (Weeks *et al*, 1998b), which is too often not discussed in the first 4–7 months after diagnosis (Huskamp *et al*, 2009). It is noteworthy that disclosure of accurate prognostic information may not be associated with the loss of hope, even in a dramatic situation (Mack *et al*, 2007).

Our analyses confirm the higher baseline cancer stage as a powerful independent prognostic factor for NSCLC mortality (Martin *et al*, 1999; Forrest *et al*, 2003). In contrast, the adjusted effect of performance status did not reach statistical significance, possibly because of limited statistical power and/or misclassification, as physicians tend to underestimate the performance status (Ando *et al*, 2001). As in other studies, smoking (Carney, 2002) and neutrophil count (Watine, 2000) were associated with increased mortality. In our study, LDH was a powerful prognostic factor: the hazard increased more than two-fold with each doubling of LDH. The effect of LDH was independent of CRP and albumin, suggesting that LDH affects survival through a biological process other than inflammation. Evidence shows that upregulation of LDH-5 is common in NSCLC and, when associated with overexpression of the Hypoxia-inducible factor 1, induces a strong anaerobic glycolytic metabolism and a reduced dependence on oxygen, resulting in decreased survival (Koukourakis *et al*, 2003). Of interest, the PH assumption was not violated for LDH, suggesting that this pathway remains unchanged over time.

Our findings regarding both CRP and albumin confirm the paramount importance of testing the assumptions underlying the very popular Cox's PH model (Altman *et al*, 1995). The striking differences between the estimated effects of both variables obtained with the Cox's PH model *vs* the flexible spline-based model are reflected in Figures 2 and 3. Overall, our results illustrate potentially serious clinical and research implications of imposing these assumptions *a priori*, which may lead to a failure to identify important prognostic factors, such as albumin in our study, inaccurate identification of high-risk groups, or spurious contradictions between the results of short- *vs* long-term prognostic studies. These results are in line with several other clinical and methodological studies indicating important violations of PH and/or linearity hypotheses (Ramsay, 1988; Sleeper and Harrington, 1990; Hastie and Tibshirani, 1990; Royston and Altman, 1994; Greenland, 1995; Rothman *et al*, 1995; Abrahamowicz *et al*, 1997; Benedetti and Abrahamowicz, 2004; Royston *et al*, 2006; Spix *et al*, 2008). The PH assumption can be tested with simple parametric or non-parametric tests available in a standard statistical software packages (Wei, 1984). If the PH hypothesis is rejected, the time-dependent effect of the prognostic factor can be estimated with flexible survival models, using either fractional polynomials (Sauerbrei *et al*, 2007) or splines (Gray, 1992; Hess, 1994; Kooperberg *et al*, 1995; Abrahamowicz *et al*, 1996; Abrahamowicz and MacKenzie, 2007), including the method incorporated in the R package (Grambsh and Therneau, 1994). To test the linearity hypothesis and estimate non-linear effects of continuous predictors on the hazard, one can use splines (Gray, 1992; Kooperberg *et al*, 1995; Abrahamowicz and MacKenzie, 2007; Remontet *et al*, 2007), or fractional polynomials (Royston and Altman, 1994; Sauerbrei *et al*, 2007; Royston and Sauerbrei, 2008), incorporated in STATA (StataCorp LP, College Station, TX, USA), R (R Foundation for Statistical Computing, Vienna, Austria) package mfp (Sauerbrei *et al*, 2006), and a SAS (SAS Institute Inc.) macro (Sauerbrei *et al*, 2006).

The flexibility of modeling offered by splines and fractional polynomials ensures that the estimated effects are represented by smooth functions rather than by ‘step-functions’, resulting from categorisation of continuous variables or of the follow-up time, which impose clinically implausible ‘jumps’ in the risk, at arbitrary selected covariate or time values. Furthermore, the flexibility of these modeling tools permits an accurate recovery of a large variety of curves with a single estimator, thus, avoiding the loss of efficiency and inaccurate statistical inference induced when the analyst uses several alternative parametric transformations of the covariate or time axis (Hastie and Tibshirani, 1990; Quantin *et al*, 1999; Mahmud *et al*, 2006).

Our study has some limitations. First, we relied on retrospective analyses of data collected prospectively on a small number of patients, for a clinical quality assessment program, in a single centre. Prospective collection increases data accuracy and reduces risk of selection or misclassification biases. However, as in other prospective studies, self-reported data on weight loss may be affected by recall bias. This might have attenuated the estimated effect of weight loss and explain its nonsignificance in the multivariable models. Second, the study population does not include all the consecutive patients observed in our POC clinic between January 2002 and January 2005 when only some physicians did test their patients for CRP. However, it is unlikely that patients of different physicians had different characteristics, because POC physicians act as a group practise. Indeed, there were no clinically relevant differences between included and excluded patients on any measured variable, except for the frequency of single *vs* double chemotherapy regimen. During the study period, double regimen was considered advantageous for the higher risk patients (Lilenbaum *et al*, 2000; Lilenbaum, 2003).

Finally, because our data are limited to a single clinical centre, the generalisability of our results and conclusions needs to be assessed in an independent study. This will also permit a direct ‘external’ validation of our flexible spline-based model. Still, the ‘internal’ cross-validation, which approximates validation in an independent sample (Harrell, 2001; Binquet *et al*, 2008), indicated that our flexible spline-based model substantially improved prediction over the Cox's PH model.

Among numerous biological markers being currently investigated (Brundage *et al*, 2002), recent reports suggest that neuron-specific enolase (NSE) may be an independent prognostic factor for survival in NSCLC (Maeda *et al*, 2000; Jacot *et al*, 2001; Ferrigno *et al*, 2003). However, NSE could not be included in our analyses, as in our institution it is used infrequently and only for diagnostic purposes (Hatzakis *et al*, 2002). Future research should assess if adjusting for NSE may affect the results of flexible analyses of NSCLC mortality.

## Conclusion

Our study has important clinical and research implications. From a research perspective, it illustrates the importance of using flexible survival models to both test the assumptions underlying the popular Cox's PH model and accurately estimate the relative risks that may change considerably during the follow-up. From a clinical perspective, it shows that while both albumin and CRP are important prognostic factors for NSCLC mortality, in this small retrospective study, their prognostic value does not extend beyond, respectively, 6 or 12 months after the initial measurement.

## Figures and Tables

**Table 1:** Patients’ characteristics (*N*=269)^a^

**Variables**	**Descriptive statistics**
Age: mean (s.d.)	64.3 (11.0)
Sex: female *n* (%)	136 (50.6)
Stage: IIIA/IIIB *n* (%)	70 (26.0)
IIIB+pleural effusion/IV *n* (%)	199 (74.0)
	
ECOG^b^ performance status:	
0–1 *n* (%)	213 (79.2)
2 *n* (%)	56 (20.8)
	
Smoking status: Never *n* (%)	41 (15.2)
Ever *n* (%)	228 (84.8)
	
Chemotherapy type:	
Single-agent *n* (%)	66 (24.5)
Double-agent *n* (%)^c^	203 (75.5)
	
Pathology:	
Adenocarcinoma *n* (%)	177 (65.8)
Large cell carcinoma *n* (%)	22 (8.2)
Squamous cell carcinoma *n* (%)	32 (11.9)
Undifferentiated carcinoma *n* (%)	38 (14.1)
	
CRP^d^:	
Mean (s.d.)	36.2 (53.6)
Median {quartile} (range)	13.1 {4.9, 39.9} (0.3, 316.8)
Log_2_ CRP:	
Mean (s.d.)	3.8 (2.2)
Median {quartile} (range)	3.7 {2.3, 5.3} (−1.7, 8.3)
	
Albumin^d^:	
Mean (s.d.)	40.2 (4.1)
	
LDH^d^:	
Mean (s.d.)	248.8 (199.4)
Median {quartile} (range)	211 {169, 263} (98, 2500)
Log_2_ LDH:	
Mean (s.d.)	7.8 (0.6)
Median {quartile} (range)	7.7 {7.4, 8.0} (6.6, 11.3)
	
Calcium^d^: mean (s.d.)	2.35 (0.19)
Alkaline phosphatase^d^: mean (s.d.)	104.2 (59.2)
Haemoglobin^d^: mean (s.d.)	130.2 (17.1)
Platelet^d^: mean (s.d.)	326.7 (117.2)
Neutrophil counts^d^: mean (s.d.)	7.09 (3.55)
Lymphocytes^d^: mean (s.d.)	1.59 (0.70)
Percentage of weight loss: mean (s.d.)	4.9 (6.6)

Abbreviations: CRP=C-reactive protein; LDH=lactate dehydrogenase.

a *N*=269 for all variables except for calcium for which *N*=264.

b Eastern cooperative oncology group.

c All include platinum-based chemotherapy.

d Normal values: CRP ⩽10 mg l^–1^; albumin 35–52 g l^–1^; LDH 110–210 U l^–1^; calcium 2.12–2.62 mmol l^–1^; alkaline phosphatase 56–120 U l^–1^; haemoglobin 140–180 g l^–1^; platelet 140–440 × 10^9^ l^–1^; neutrophil counts 1.60–7.70 × 10^9^ l^–1^; and lymphocytes 0.80–4.40 × 10^9^ l^–1^.

**Table 2:** Results of univariate Cox's PH models (*N*=269)^a^

**Variables**	**HR (95% CI)^b^**	***P*-value for test of no association**	***P*-value for test of PH**	***P*-value for test of linearity**
Age	1.009 (0.996, 1.021)	0.180	0.119	0.268
Sex: (male *vs* female)	1.458 (1.109, 1.917)	0.007	0.817	N/A
Stage: (IIIB+pleural effusion/4 *vs* IIIA/IIIB)	2.031 (1.454, 2.837)	<0.001	0.426	N/A
ECOG^c^ performance status: (2 *vs* 0–1)	2.034 (1.488, 2.782)	<0.001	0.047	N/A
Smoking status: (ever *vs* never)	1.934 (1.278, 2.927)	0.002	0.253	N/A
Chemotherapy type: (single *vs* double)	1.845 (1.353, 2.515)	<0.001	0.153	N/A
Pathology: adenocarcinoma	Ref	Ref	Ref	N/A
Large cell carcinoma	1.072 (0.670, 1.716)	0.771	0.124	
Squamous cell carcinoma	0.934 (0.611, 1.428)	0.753	0.610	
Undifferentiated carcinoma	1.283 (0.858, 1.917)	0.225	0.193	
Log_2_ CRP: (per doubling of CRP values)	1.175 (1.102, 1.252)	<0.001	0.002	0.078
Albumin: (per ↓^d^ of 1 g l^–1^)	1.098 (1.061, 1.136)	<0.001	<0.001	0.190
Log_2_ LDH: (per doubling of LDH values)	2.336 (1.877, 2.909)	<0.001	0.130	0.093
Calcium: (per ↑^e^ of 1 mmol l^–1^)	2.025 (0.897, 4.571)	0.089	0.533	0.137
Alkaline phosphatase: (per ↑ of 10 U l^–1^)	1.044 (1.020, 1.068)	<0.001	0.443	0.930
Haemoglobin: (per ↓ of 10 g l^–1^)	1.094 (1.009, 1.186)	0.030	0.087	0.394
Platelet: (per ↑ of 10 × 10^9^ l^–1^)	1.011 (0.999, 1.023)	0.063	0.044	0.623
Neutrophil counts: (per ↑ of 1 × 10^9^ l^–1^)	1.117 (1.071, 1.164)	<0.001	0.010	0.030
Lymphocytes: (per ↓ of 1 × 10^9^ l^–1^)	1.490 (1.214, 1.829)	<0.001	0.132	0.702
Percentage of weight loss: (per ↓ of 1%)	1.020 (1.000, 1.041)	0.049	0.157	0.204

Abbreviations: CRP=C-reactive protein; LDH=lactate dehydrogenase; PH=proportional hazard.

N/A: the test of linearity is not applicable to categorical covariates.

a *N*=269 for all variables except for calcium for which *N*=264.

b Unadjusted hazard ratio (HR) and 95% confidence interval (95% CI).

c Eastern cooperative oncology group.

d ↓: decrease.

e ↑: increase.

**Table 3:** Results of the multivariable Cox's PH model (*N*=269)

**Variables**	**HR (95% CI)^a^**	***P*-value for test of no association**	***P*-value for test of PH**	***P*-value for test of linearity**
Stage: (IIIB+pleural effusion/4 *vs* IIIA/IIIB)	1.815 (1.268, 2.597)	0.001	0.204	N/A
ECOG^b^ performance status: (2 *vs* 0-1)	1.348 (0.958, 1.896)	0.086	0.165	N/A
Smoking status: (ever *vs* never)	2.087 (1.349, 3.230)	0.001	0.135	N/A
Chemotherapy type: (single *vs* double)	1.539 (1.082, 2.188)	0.016	0.067	N/A
Log_2_ CRP: (per doubling of CRP values)	1.108 (1.027, 1.196)	0.008	0.039	0.130
Albumin: (per ↓^c^ of 1 g l^–1^)	1.015 (0.974, 1.058)	0.485	<0.001	0.024
Log_2_ LDH: (per doubling of LDH values)	2.159 (1.700, 2.742)	<0.001	0.636	0.590
Alkaline phosphatase: (per ↑^d^ of 10 U l^–1^)	1.019 (0.993, 1.047)	0.150	0.075	0.034
Neutrophil counts: (per ↑ of 1 × 10^9^ l^–1^)	1.082 (1.037, 1.129)	<0.001	0.027	0.041
Lymphocytes: (per ↓ of 1 × 10^9^ l^–1^)	1.307 (1.050, 1.626)	0.016	0.550	0.460
Deviance^e^	1902.2			
AIC	1922.2			

Abbreviations: AIC=Akaike's information criterion; CRP=C-reactive protein; LDH=lactate dehydrogenase; PH=proportional hazard.

N/A: the test of linearity is not applicable to categorical covariates.

a Adjusted hazard ratio (HR) and 95% confidence interval (95% CI).

b Eastern cooperative oncology group.

c ↓: decrease.

d ↑: increase.

e Deviance=−2^*^log-likelihood.

**Table 4:** Results of the flexible spline-based model (*N*=269)

**Variables**	**HR (95% CI)^a^**	***P*-value for test of no association**
Stage: (IIIB+pleural effusion/4 *vs* IIIA/IIIB)	1.859 (1.284, 2.691)	<0.001
ECOG^b^ performance status: (2 *vs* 0–1)	1.336 (0.923, 1.935)	0.116
Smoking status: (ever *vs* never)	2.248 (1.419, 3.561)	<0.001
Chemotherapy type: (single *vs* double)	1.462 (0.990, 2.160)	0.041
Log_2_ CRP: (per doubling of CRP values)	^*^	0.003 (overall *P*-value)^#^
Albumin: (per ↓^c^ of 1 g l^–1^)	^**^	0.001 (overall *P*-value)^#^
Log_2_ LDH: (per doubling of LDH values)	2.281 (1.661, 3.142)	<0.001
Alkaline phosphatase: (per ↑of 10 U l^–1^)	1.012 (0.980, 1.041)	0.366
Neutrophil counts: (per ↑^d^ of 1 × 10^9^ l^–1^)	1.072 (1.025, 1.122)	0.001
Lymphocytes: (per ↓ of 1 × 10^9^ l^–1^)	1.313 (1.035, 1.666)	0.012
Deviance^e^	1873.3	
AIC	1909.3	

Abbreviations: AIC=Akaike's information criterion; CRP=C-reactive protein; LDH=lactate dehydrogenase.

a Adjusted hazard ratio (HR) and 95% confidence interval (95% CI).

b Eastern cooperative oncology group.

c ↓: decrease.

d ↑: increase.

e Deviance=−2^*^log-likelihood.

^*^Both the time-dependent (*P*=0.033) and non-linear (*P*=0.015) effects were significant. The estimated non-linear effects, at selected follow-up times, are shown in Figure 2.

^**^Both the time-dependent (*P*=0.0001) and non-linear (*P*=0.038) effects were significant. The estimated non-linear effects, at selected follow-up times, are shown in Figure 3.

^#^*P*-value for a likelihood ratio test, with 5 degrees of freedom, of the null hypothesis of no association, obtained by comparing the deviances of (i) a flexible model where both time-dependent and non-linear effects of a given variable are modeled by splines, *vs* (ii) a simpler ‘reduced’ model, which does not include the variable being tested (see the section on “Statistical analyses” for details of the test).
